# Longer incubation period of coronavirus disease 2019 (COVID‐19) in older adults

**DOI:** 10.1002/agm2.12114

**Published:** 2020-05-22

**Authors:** Tak‐kwan Kong

**Affiliations:** ^1^ Department of Medicine & Therapeutics Prince of Wales Hospital The Chinese University of Hong Kong Hong Kong SAR China

**Keywords:** COVID‐19, incubation period, older adults

## Abstract

**Objective:**

The aim of this study was to explore any age‐related change in the incubation period of COVID‐19, specifically any difference between older (aged ≥65 years) and younger adults.

**Methods:**

Based on online data released officially by 21 Chinese cities from January 22 to February 15, 2020, the incubation period of COVID‐19 patients who had travelled to Hubei was studied according to age. Previous studies were reviewed and compared.

**Results:**

The study recruited 136 COVID‐19 patients who had travelled to Hubei during January 5‐31, 2020, stayed for 1‐2 days, and returned with symptom onset during January 10‐February 6, 2020. The median age was 50.5 years (range 1‐86 years), and 22 patients (16.2%) were aged ≥65 years. The age‐stratified incubation period was U‐shaped with higher values at extremes of age. The median COVID‐19 incubation period was 8.3 (90% confidence interval [CI], 7.4‐9.2) days for all patients, 7.6 (90% CI, 6.7‐8.6) days for younger adults, and 11.2 (90% CI, 9.0‐13.5) days for older adults. The 5th/25th/75th/90th percentiles were 2.3/5.3/11.3/14.2 days for all, 2.0/5.0/10.5/13.2 days for younger adults, and 3.1/7.8/14.4/17.0 days for older adults. There were 11 published studies on COVID‐19 incubation periods up to March 30, 2020, reporting means of 1.8‐7.2 days, and medians of 4‐7.5 days, but there was no specific study on the effect of age on incubation period. One study showed that severe COVID‐19 cases, which included more elderly patients, had longer incubation periods.

**Conclusion:**

Based on 136 patients with a travel history to Hubei, the epicenter of COVID‐19, the COVID‐19 incubation period was found to be longer in older adults. This finding has important implications for diagnosis, prevention, and control of COVID‐19.

## 1 INTRODUCTION

Coronavirus disease 2019 (COVID‐19), caused by the emergence of the novel pathogen severe acute respiratory syndrome coronavirus 2 (SARS‐CoV‐2), has spread with enormous speed and scale since its first reports in Wuhan, Hubei Province, China, in late December 2019.^1^ A pandemic was declared by the World Health Organization (WHO)^2^ on March 11, 2020. As of April 5, 2020, the reported cumulative number of confirmed patients had reached 1 133 758 globally in over 100 countries and territories, associated with 62 784 deaths.^3^

Older adults are at higher risk of contracting COVID‐19, developing a severe disease, and dying from the disease,^4^, ^5^, ^6^ similar to what was experienced during the severe acute respiratory syndrome (SARS) epidemic in 2003.^7^, ^8^ In China, 90% of the COVID‐19 deaths occurred in those older than 60 years, and 20% of all deaths were people aged older than 80 years.^4^ In Europe, where the proportion of elderly population (19% aged over 65 years) is higher, the impact is greater: over 95% of the COVID‐19 deaths has occurred in those aged over 60 years, and more than 50% of all deaths have been people aged over 80 years.^6^ Because of immune senescence and common occurrence of secondary immunodeficiency in old age, the presentations and disease course of older adults in response to infectious disease may be altered.^9^, ^10^

A review of SARS in old age^7^ notes that, according to clinical experience, its incubation period, defined as the time between infection exposure and symptom onset, is apparently longer in older adults, but there is a lack of studies on the incubation periods of SARS‐associated coronavirus, specifically in older adults. Knowing the incubation period of this novel virus is relevant for diagnosis, surveillance, prevention, and control of COVID‐19, and it is important to know whether its incubation period in older adults, who represent a vulnerable group to this disease, differs from that in younger adults. The objective of this study was therefore to explore whether there is any age‐related change in the incubation period of COVID‐19, specifically any difference between older (aged ≥65 years) and younger adults.

## 2 PATIENTS AND METHODS

### 2.1 Data source and collection

For nearly every city in China, daily information on the list of COVID‐19 cases is released officially to the Chinese social media WeChat accounts of respective cities. However, only a minority of cities include in their official release clear information on the day of symptom onset, which is required in estimating incubation period. The information released between January 22 and February 15, 2020 was captured and compiled into a list of patients with COVID‐19 from cities that reported the day of symptom onset and those who had travelled to Hubei, the epicenter of the COVID‐19 epidemic. The following data were entered into an Excel (Microsoft) spreadsheet for COVID‐19 cases reported between January 22 and February 15, 2020 from 21 Chinese cities outside Hubei: patient case number, age, sex, first day to Hubei, last day in Hubei, and first day with symptoms. The data were collected by another person acknowledged at the end of this paper.

### 2.2 Data analysis

For this study, only those COVID‐19 patients who stayed in Hubei for at most two calendar days were included. The day of exposure was taken as the first day to Hubei if the patient had stayed in Hubei for one calendar day; or as the middle of the first day and second day in Hubei if the patient had stayed for two calendar days. By excluding COVID‐19 patients who had stayed in Hubei for more than 2 days, a narrowly defined exposure window was ensured, thus reducing the uncertainty in estimating the day of exposure. The incubation period for each COVID‐19 patient was derived from the number of days between exposure and symptom onset. Parametric and non‐parametric methods were used in the statistical analysis. The COVID‐19 incubation periods for different age groups of 15‐year interval size were analyzed in terms of their respective mean, median, 25th, and 75th percentiles. Next, the frequency distributions of COVID‐19 incubation periods of the three age groups—children (age 0‐14 years), younger adults (age 15‐64 years), and older adults (aged ≥65 years)—were plotted and compared. The cumulative frequency distributions for the incubation periods for both younger and older adult groups were analyzed using the cumulative frequency analysis software CumFreq.^11^ This computer program fits the observed cumulative frequency data into a best‐fit theoretical distribution by the regression method. Observed data are plotted in ranked order of increasing probability, and the most appropriate theoretical distribution is selected based on the lowest average of absolute values of the differences between observed and calculated cumulative frequency values. Further details are available at the CumFreq website.^11^ With the use of CumFreq, probability distribution curve fitting was performed for the incubation periods for both younger and older adult groups, and their medians, quartiles, 5th, and 90th percentiles estimated.

### 2.3 Literature review

The author searched the studies reporting on incubation periods of COVID‐19 archived in PubMed (published) and medRxiv (unpublished) until March 30, 2019. The following search terms were used: (“incubation”) and (“COVID‐19” or “SARS‐CoV‐2”). Studies reporting on the incubation period of COVID‐19 were included for further evaluation and comparison with this study. Information on study place, sample size, mean/median age, percentage of subjects studied who were elderly, and incubation period was extracted from these previous studies.

## 3 RESULTS

A total of 136 COVID‐19 patients were recruited into this study. They had travelled to Hubei from 21 Chinese cities between January 5 and January 31, 2020, had stayed there for at most two calendar days, and had returned to their respective cities with symptom onset between January 10 and February 6, 2020. The median age was 50.5 years (range 1‐86 years, interquartile range, 36.8‐60.5 years), 22 (16.2%) patients were aged 65 years or above, and 72 (53%) patients were male. By parametric tests, the mean incubation period of the 136 patients was 8.5 days (95% confidence interval [CI], 7.8‐9.2 days), with a median of 8.3 days (95% CI, 7.6‐9.0 days) and an interquartile range of 4.9‐12.0 days.

**Figure 1:**
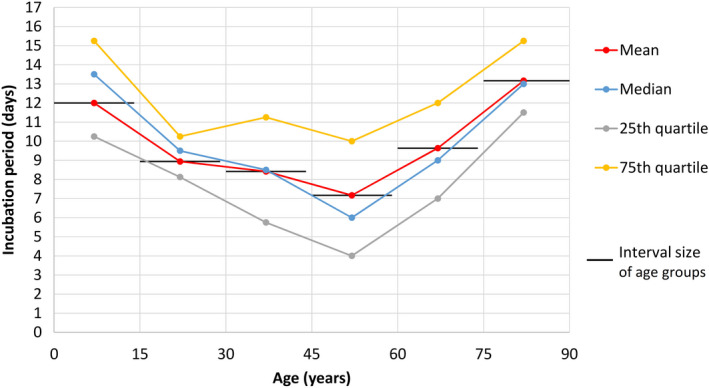
COVID‐19 incubation period according to age

When plotted against age with group size of 15 years, the incubation period showed a U‐shaped curve with higher values at extremes of age (Figure 1). The mean/median incubation period values dropped from 12.0/13.5 days for age 0‐14 years (n = 4) to 8.9/9.5 days for age 15‐29 years (n = 8), 8.4/8.5 days for age 30‐44 years (n = 36), 7.2/6.0 days for age 45‐59 years, and then rose to 9.6/9.0 days for age 60‐74 years (n = 29) and 13.2/13.0 days for age 75‐89 years (n = 6).

**Figure 2:**
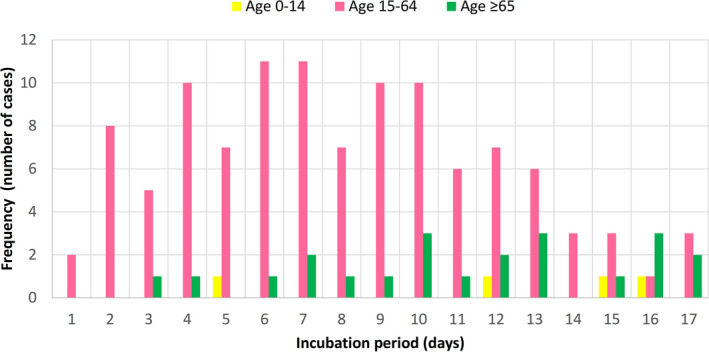
Frequency distribution of COVID‐19 incubation period for older adults (aged ≥65 years), younger adults (aged 15‐64 years), and children (aged 0‐14 years)

**Figure 3:**
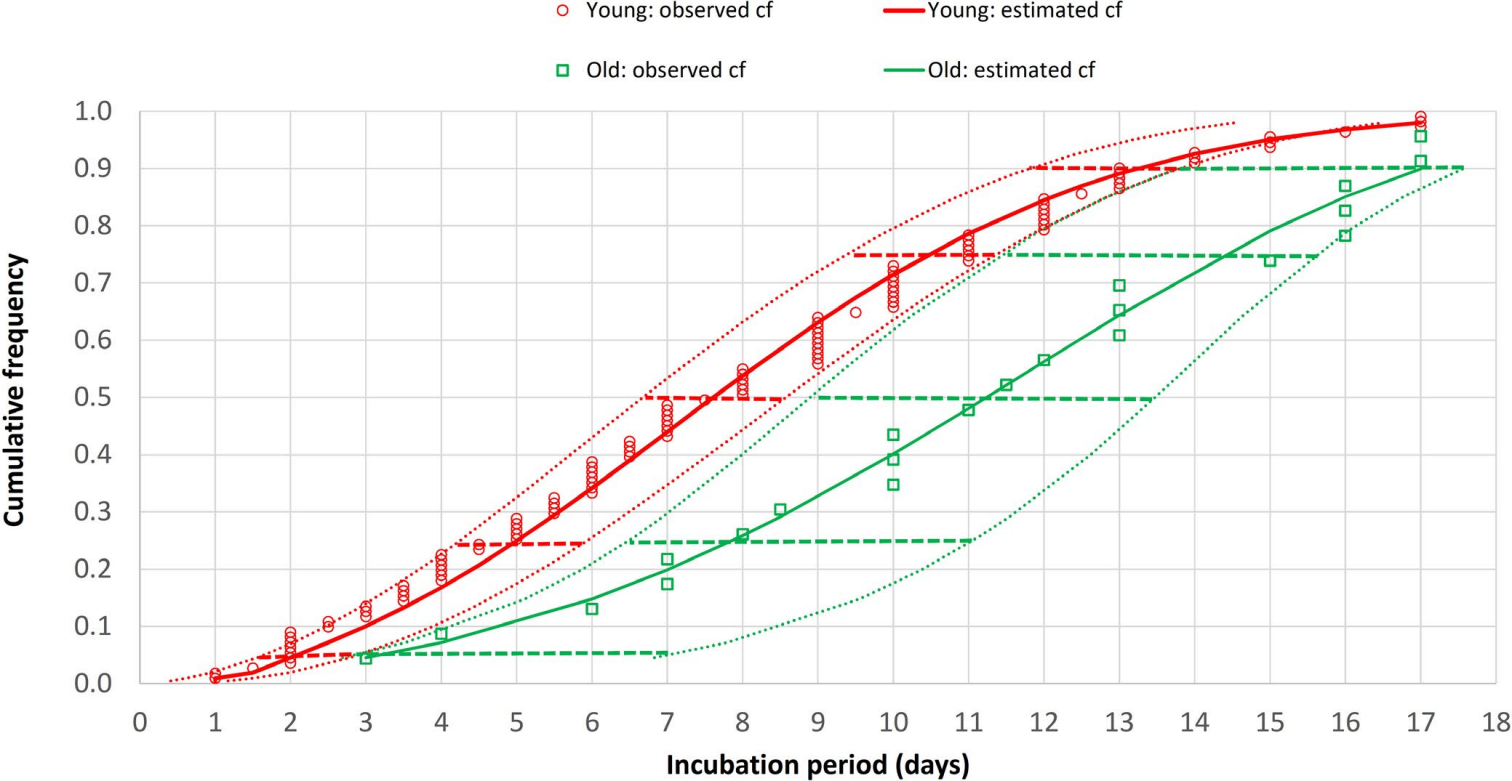
Cumulative frequency (cf) distribution of COVID‐19 incubation period for older (aged ≥65 years) and younger (aged 15‐64 years) adults. Dotted curves indicate the 90% confidence intervals (CIs) of the incubation period distribution while horizontal bars represent the 90% CIs of the percentiles (5th, 25th, 50th [median], 75th, and 90th)

There were 22 patients aged ≥65 years, 110 patients aged 15‐64 years, and four patients aged 0‐14 years in this study. Their frequency distributions of COVID‐19 incubation periods did not fit into a normal pattern (Figure 2). There was a skew towards the left for younger adults, but for older adults, a skew towards the right with wider variation was noted. Cumulative frequency distributions of the entire study population, the younger (15‐64 years), and older (≥65 years) adult age groups were further analyzed by the CumFreq^11^ software (Figure 3). Kumaraswamy distribution returned as the best fit for the entire study population and the younger adult group, while the mirrored generalized Gumbel (also known as the log‐Weibull distribution) returned as the best fit for the older adult group. This gave estimates of the non‐parametric values of the COVID‐19 incubation period for the total study population, and the younger and older adult age groups, as shown in Table 1. The medians were 8.3 (90% CI, 7.4‐9.2) days for all patients, 7.6 (90% CI, 6.7‐8.6) days for younger adults, and 11.2 (90% CI, 9.0‐13.5) days for older adults. The 5th/25th/75th/90th percentiles were 2.3/5.3/11.3/14.2 days for all patients, 2.0/5.0/10.5/13.2 days for younger adults, and 3.1/7.8/14.4/17.0 days for older adults.

**Table 1:** Present study on COVID‐19 incubation period

Age groups (years)	Case number studied (%)	Median age in years (range)	Incubation period in days (90% CI)		
			Estimated medians	Estimated percentiles	Observed ranges
All	136	50.5 (1‐86); IQR 36.8‐60.5	8.3 (7.4‐9.2)	P5 = 2.3 (1.7‐3.0), P25 = 5.3 (4.6‐6.3), P75 = 11.3 (10.3‐12.1), P90 = 14.2 (12.8‐14.6)	1‐17
15‐64	110 (80.9%)	49.0 (20‐64)	7.6 (6.7‐8.6)	P5 = 2.0 (1.6‐2.9), P25 = 5.0 (4.2‐5.9), P75 = 10.5 (9.4‐11.3), P90 = 13.2 (11.8‐13.8)	1‐17
≥65	22 (16.2%)	69 (65‐86)	11.2 (9.0‐13.5)	P5 = 3.1 (2.9‐7.0), P25 = 7.8 (6.5‐11.0), P75 = 14.4 (11.5‐15.6), P90 = 17.0 (13.8‐17.6)	3‐17

Abbreviations: CI, confidence interval; IQR, interquartile range; Pn, nth percentile.

The literature review retrieved 11 published^5^, ^12^, ^13^, ^14^, ^15^, ^16^, ^17^, ^18^, ^19^, ^20^, ^21^ and two unpublished^22^, ^23^ studies that reported on COVID‐19 incubation periods. Their details are listed in Table 2.

**Table 2:** Previous studies on COVID‐19 incubation period

Author	Place	Case number studied/parent sample	Median age in years (range)	Number (%) aged ≥ 65 years in studied/parent sample	Incubation period in days		
					Mean (95% CI)	Median (95% CI)	Percentiles (95% CI) & range
Li et al^12^	Wuhan, China	10/425	59 (15‐89)	NA/162 (38%)	5.2^a^ (4.1‐7.0)		P95^a^ = 12.5 (9.2‐18)
Sun et al^5^	China and countries outside China (including travelers to Wuhan)	33/507	46 (IQR 35‐60)	NA/96 (18.9%)		4.5	IQR = 3.0‐5.5, R = 1‐9.5
Guan et al^13^	China (30 provinces)	291/1099	47 (IQR 35‐58)	NA/166 (15.1%)		4	IQR = 2‐7
Backer et al^14^	Wuhan, China (travelers)	88	42 (2‐72)	11 (13.1%)	6.8^a^ (5.7‐8.8), 6.4^b^ (5.6‐7.7)		P5^a^ = 2.8 (2.0‐3.5), P95^a^ = 13.3 (9.9‐20.5)
Jiang et al^15^	China	50	NA	NA	4.9 (4.4‐5.5)		
Lauer et al^16^	China and 17 countries outside Hubei	101	52 (IQR 36.5‐59)	16 (15.8%)	5.5^a^	5.2^a^ (4.4‐6.0)	P2.5^a^ = 2.5 (1.8‐3.6), P97.5^a^ = 10.5 (7.3‐15.3)
Linton et al^17^	Wuhan (residents, travelers)	158	Most 30‐59	NA	5.6^a^ (5.0‐6.3)	5.0^a^ (4.4‐5.6)	P95^a^ = 10.6 (8.5‐14.1)
Qian et al^18^	Zhejiang, China (including travelers to Wuhan)	91	50 (IQR 36.5‐57; range 5‐96)	6 (6.6%) for age ≥70		6	IQR = 3‐8
Leung^19^	China outside Hubei (including travelers to Hubei)	105 (travelers)	Mean 41.2		1.8^b^ (1.0‐2.7)		P95^b^ = 3.2 (1.0‐3.8)
		70 (non‐travelers)			7.2^b^ (6.1‐8.4)		P95^b^ = 14.6 (12.1‐17.1)
Tian et al^20^	Beijing, China (including travelers to Wuhan)	262 (all)	47.5 (1‐94)	48 (18.3%)		6.7 ± 5.2	
		46 (severe)	61.4 (1‐94)	20 (43.5%)		7.5 ± 7.2	
		216 (milder)	44.5 (1‐93)	28 (13%)		6.5 ± 4.6	
Cai et al^21^	Shanghai, China	10	Mean 6.2 (0.25‐10.9)	0%	6.5		R = 2‐10
Jing et al^22^, c	China and countries outside Hubei (travelers to Wuhan)	1211/12 963	40 (0.5‐86)	160 (13.2%)/2554 (19.7%) for age ≥60	8.62^b^ (8.02‐9.28)	8.13^b^ (7.37‐8.91)	P90^b^ = 14.65 (14.00‐15.26), P99^b^ = 20.59 (19.47‐21.62)
Tindale et al^23^, c	Singapore	93	NA	NA	7.2^a^ (6.2‐8.3), 7.1^b^ (6.1‐8.3)	6.55^b^	
	Tianjin, China	125	NA	NA	9.3^a^ (7.9‐10.9), 9.0^b^ (7.9‐10.2)	8.62^b^	

Abbreviations: CI, confidence interval; IQR, interquartile range (25th to 75th percentiles); NA, not available; Pn, nth percentile; R, range (0th to 100th percentiles),

^a^ By lognormal fit.

^b^ By Weibull fit.

^c^ Unpublished.

## 4 DISCUSSION

The first estimate of the COVID‐19 incubation period was reported by Li et al^12^ as a mean of 5.2 days (95% CI, 4.1‐7.0) and a 95th percentile of 12.5 days (95% CI, 9.2‐18), based on their study on the exposure information of 10 confirmed COVID‐19 patients in Wuhan, China (Table 2). This is slightly longer than the incubation period estimated for SARS, with a median of 4.0 days (95% CI, 3.6‐4.4) and a 95th percentile of 10.6 days (95% CI, 8.9‐12.2).^24^ Subsequent published studies^5^, ^12^, ^13^, ^14^, ^15^, ^16^, ^17^, ^18^, ^19^, ^20^, ^21^ on the COVID‐19 incubation period (Table 2) have reported means varying from 1.8 days to 7.2 days, medians of 4‐7.5 days, and 95th percentiles of 3.2‐14.6 days, which may be due to differences in methodologies and patient samples.

This study of 136 COVID‐19 patients revealed a longer incubation period, with a mean of 8.5 days, an estimated median of 8.3 days, and an interquartile range of 5.3‐11.3 days. Interestingly, two unpublished studies^22^, ^23^ also reported longer COVID‐19 incubation periods with mean/median of 8.62/8.13 days and 9.0/8.62 days, respectively (Table 2). The longer incubation period reported by Jing et al,^22^ who studied a large sample size of 1211 individuals who had been asymptomatic at their time of departure from Wuhan, may be related to the forward follow‐up methodology of a sufficiently long duration of 25 days until symptoms had developed so that those with longer incubation periods were not missed from sample collection. Tindale et al^23^ attributed their finding of longer incubation period to missed intermediate exposure events due to pre‐symptomatic transmission and misperceived exposure times.

In this study, the incubation period of COVID‐19, when age‐stratified, displayed a U‐shaped curve with higher values at the extremes of age for the pediatric and geriatric age groups (Figure 1). A study by Cai et al^21^ of COVID‐19 in 10 children (mean age, 6.2 years; range 0.25‐10.9 years) also reported a longer incubation period of 6.5 days in this age group (Table 2). The small sample size of four in the present study’s child group (age 0‐14 years) prevented further statistically significant analysis. With this U‐shaped age‐related finding of COVID‐19 incubation period, as well as the background of an apparent longer incubation period for SARS in old age from clinical experience in 2003,^7^ and also a reported case of a 70‐year‐old COVID‐19 patient with a long incubation period of 27 days in Hubei,^25^ the author proceeded to analyze the incubation period of COVID‐19 in older adults compared with younger adults.

The frequency distribution of COVID‐19 incubation period for 22 older adults (aged ≥65 years) was compared with that of 110 younger adults (aged 15‐64 years). From the frequency distributions of incubation periods for the two age groups, the younger adult group skewed towards the left (shorter incubation period) while the older adult group skewed towards the right (longer incubation period). Neither of the frequency distributions fit into a normal pattern (Figure 2). Sartwell^26^ showed that for most infectious diseases, the frequency curves of the incubation period take the form of logarithmic normal distribution. Based on this observation, lognormal fitting of distribution data has been used to estimate the incubation period parameters of common respiratory viral infections.^24^ However, this lognormal fitting has been challenged in the review by Nishiura,^27^ who noted lack of validity in assuming lognormal distribution and the need for using different distributions to compare for best fit. Previous studies on COVID‐19 have also employed probability distribution fitting methods other than lognormal and some have come up with better fits (Table 2). Using the ComFreq software for cumulative frequency distribution fitting for patients of this study, Kumaraswamy distribution returned as the best fit for the younger adult group, while the mirrored generalized Gumbel (also known as the log‐Weibull distribution) returned as the best fit for the older adult group; the latter also displayed wider 90% confidence limits (Figure 3). Ninety percent instead of 95% confidence limits were used for the analysis because of the smaller sample size of 22 older adults. This gave estimates of the non‐parametric values of the COVID‐19 incubation period for the two age groups (Table 1). The estimated median, interquartile range, and 90th percentile of the COVID‐19 incubation period for older adults were 11.2, 7.8‐14.4, and 17.0 days, respectively, which are longer than the corresponding figures of 7.6, 5.0‐10.5, and 13.2 days for younger adults. The respective 90% confidence intervals of the incubation period distributions for younger and older groups did not overlap despite of the wider 90% confidence limits of the older group.

**Figure 4:**
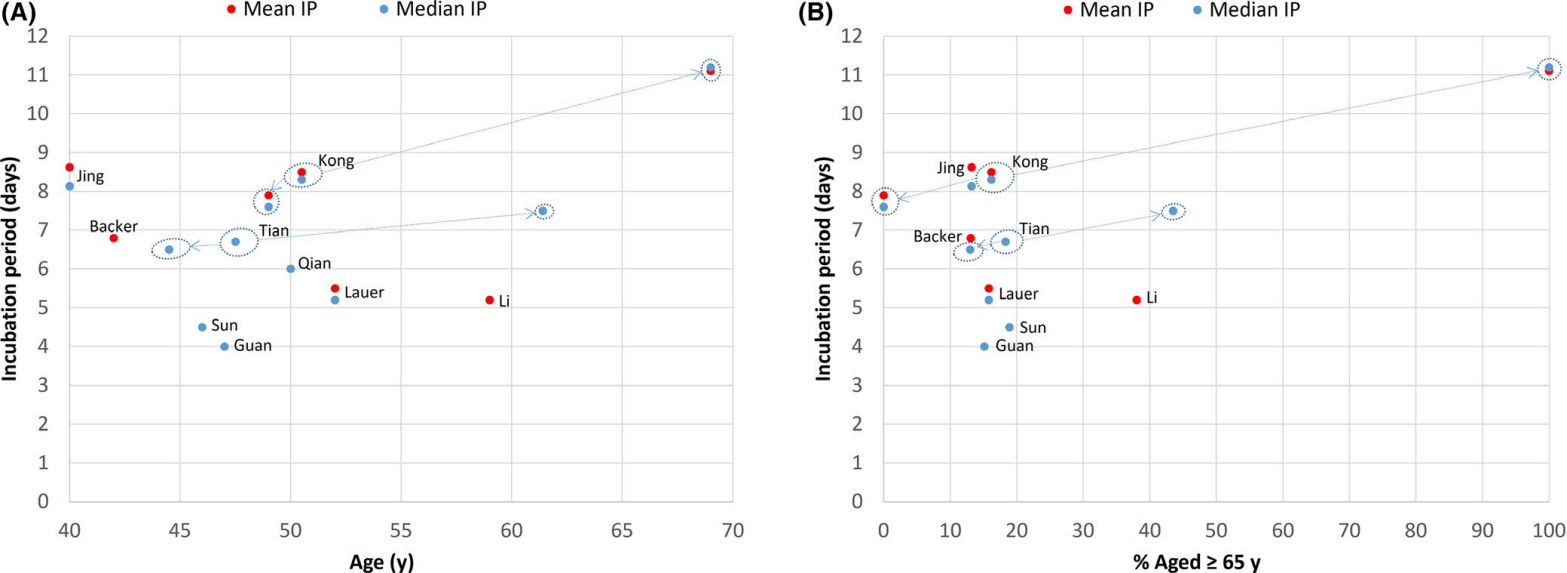
COVID‐19 incubation period (IP) according to age in previous and present studies: (A) mean or median age, (B) percentage aged ≥65 years. Substudies are indicated by arrows from main study. “Age” refers to age of study sample of incubation period when available, or to age of parent sample when age is not available for study sample

Although previous studies on COVID‐19 incubation period did not look specifically at the effect of older age on incubation period, I reviewed these studies for any such clues. Of the ten published studies^5^, ^12^, ^13^, ^14^, ^15^, ^16^, ^17^, ^18^, ^19^, ^20^ on COVID‐19 incubation period (Table 2) that included adults, eight had information on mean or median age. Except for the first study by Li et al,^12^ which included much older subjects in the parent population but without age information for the 10 patients studied in the incubation period subset, the remaining seven adult studies^5^, ^13^, ^14^, ^16^, ^18^, ^19^, ^20^ had median age of 42‐52 years with around 15% aged over 65 years, which is not too dissimilar to this study population (median age, 50.5 years; 16.2% aged over 65 years). However, there was wide variation in the incubation period values reported from these studies (Figure 4), which may be due to differences in methodologies and patient samples. These factors, together with a lack of detailed information on the age structure and the small number of older adults recruited, made direct comparison with this study difficult. Nevertheless, when the clinical and epidemiological study of COVID‐19 by Tian et al^20^ in Beijing was reviewed (Figure 4), it was noted that the incubation period for the severe group (whose subjects were older with a median age of 61.4 years and 43.5% aged over 65 years) had a longer median incubation period of 7.5 ± 7.2 days when compared with 6.5 ± 4.6 days for the milder group (whose subjects were younger with a median age of 44.5 years and 13% aged over 65 years). The unpublished study by Jing et al^22^ reports on a long COVID‐19 incubation period (mean, 8.62 days; median, 8.13 days) that is close to the present figures, but their sample was younger (median age 40 years; 13.2% aged over 60 years) and a different methodology was used as discussed above.

The incubation period of an infection is influenced by infectious dose and immune response. Thus, the shorter COVID‐19 incubation period among travelers to Hubei compared with non‐travelers in the study by Leung^19^ was attributed to exposure to a higher viral load among travelers to Hubei, the epicenter. The longer COVID‐19 incubation period observed in this study for older adults may be accounted for by the blunted immune response due either to age‐associated immune senescence or secondary immunodeficiency in old age.^7^, ^9^, ^10^ The lack of a fever response in elderly subjects, the non‐specific geriatric presentations in an infectious illness (such as falls and delirium), and multi‐comorbidities might result in a delayed awareness of disease onset and its detection by a clinician.^6^ Multiple exposures, instead of a well‐defined exposure event, might occur for frailer elderly people because of their need for close personal and nursing care. Though this latter point may be less relevant for subjects of this study, whose exposure time was narrowed to within two calendar days of travel to Hubei and who were likely to be fitter because of their ambulatory status, it is nevertheless relevant in the setting of long‐term care facilities and hospital wards. Multiple contact dates have been shown to be associated with longer incubation periods of over 10 days in SARS using computer simulation.^28^

The finding in this study of longer COVID‐19 incubation period for older patients has several implications for clinical practice, public‐health policy, and research on this disease. Knowing the incubation period of an infectious disease aids in its detection and diagnosis. Knowledge that an older adult can have a longer COVID‐19 incubation period will minimize underdiagnosis and increase suspicion despite atypical presentations. Delayed diagnoses of SARS and COVID‐19 in older adults were associated with outbreaks in nursing homes and long‐term care facilities.^7^, ^29^ A longer 90th percentile of 17 days (90% CI, 13.9‐17.6 days) for older adults means that 10% of older subjects would have a COVID‐19 incubation period of over 17 days. Figure 3 shows that only 72% of older adults had a COVID‐19 incubation period of ≤14 days, compared with 93% for younger adults. Thus, while the current quarantine period of 14 days that has been adopted for surveillance, prevention, and control of COVID‐19 might appear adequate for younger adults (7% fell outside this period), this would be inadequate for older adults (28% fell outside this period). Older adults would therefore require a longer period of isolation and observation; extending the quarantine period from 14 to 17 days would increase the coverage from 72% to 90%. A longer incubation period may also be associated with a higher rate of asymptomatic, pre‐symptomatic, and subclinical infection towards the end of the long incubation period.^23^ Asymptomatic and pre‐symptomatic SARS‐CoV‐2 infections have been described in half of the 23 residents (mean age, 80.7 years) who tested positive for COVID‐19 during an outbreak at a long‐term‐care skilled‐nursing facility in Washington State, United States.^30^ While there has been no documented asymptomatic transmission,^31^ pre‐symptomatic transmission (transmission of COVID‐19 during incubation period before symptom onset) has been documented during contact tracing and investigation of infection clusters.^32^, ^33^

The strengths of this study are, first, a clear exposure day by sampling travelers who had stayed in Hubei for at most two calendar days and, second, the inclusion of enough older adults to compare their incubation period with younger adults. Previous studies^5^, ^14^, ^17^, ^19^ based on traveler history to Hubei included cases with longer and imprecise exposure period. Except for two studies,^20^, ^22^ the number of older adults aged over 65 recruited was either unknown or too small (6‐11) to allow age comparison.

The limitations of this study are, first, data sourced online instead of clinically. Online data tracking on travel histories and dates of symptom onset to estimate COVID‐19 incubation periods have also been used in seven of the previous studies.^5^, ^14^, ^16^, ^17^, ^19^, ^22^, ^23^ These and the present study assumed that COVID‐19 infection was acquired during travel to Hubei, the epicenter. This may not be true, especially during late January 2020 when the infection had spread to other cities of China. Thus, travelers to Hubei may have acquired the infection on return to their own cities so that the incubation period was overestimated if based on staying in Hubei as infection exposure. Second, elderly people aged ≥65 years do not form a homogenous group in terms of health status. The data collected in this study did not have clinical information on frailty, which may be associated with multiple exposures, blunted immune response, and geriatric presentations that impact on incubation period estimates. On the other hand, older subjects recruited into this study may be biased towards the fitter spectrum because of their traveler status. Nevertheless, the distribution of COVID‐19 incubation period for older adults in this study had a wide variation. There is uncertainty if the wide variation observed arose from heterogeneity in the fitness‐frailty spectrum, a hallmark of aging, or because of the relatively small number of older adults sampled. Further studies involving a larger sample size of older adults and frailty statuses may help to clarify this. Third, the study sample was drawn from a listing of hospitalized individuals. Thus, milder community cases of COVID‐19 were not represented in this study.

## CONFLICTS OF INTEREST

Nothing to disclose.

## AUTHOR CONTRIBUTIONS

The author is responsible for design, conceptualization, literature review, data analysis, and writing of paper.
