# CORRIGENDUM

**DOI:** 10.1002/agm2.12129

**Published:** 2020-11-04

In Volume 3, Issue 2 (2020),^1^ updates to references 22 and 23 and changes to the values of incubation periods quoted to reflect the updated values in references 22 and 23, which have been converted from preprints to published articles, are required.

The updated text and references are below:

**Abstract**

**Results**

The study recruited 136 COVID‐19 patients who had travelled to Hubei during January 5‐31, 2020, stayed for 1‐2 days, and returned with symptom onset during January 10‐February 6, 2020. The median age was 50.5 years (range 1‐86 years), and 22 patients (16.2%) were aged ≥65 years. The age‐stratified incubation period was U‐shaped with higher values at extremes of age. The median COVID‐19 incubation period was 8.3 (90% confidence interval [CI], 7.4‐9.2) days for all patients, 7.6 (90% CI, 6.7‐8.6) days for younger adults, and 11.2 (90% CI, 9.0‐13.5) days for older adults. The 5th/25th/75th/90th percentiles were 2.3/5.3/11.3/14.2 days for all, 2.0/5.0/10.5/13.2 days for younger adults, and 3.1/7.8/14.4/17.0 days for older adults. There were 13 published studies on COVID‐19 incubation periods up to March 30, 2020, reporting means of 1.8‐8.68 days, and medians 4‐8.06 days, but there was no specific study on the effect of age on incubation period. One study showed that severe COVID‐19 cases, which included more elderly patients, had longer incubation periods.

**3. RESULTS**

The literature review retrieved 13 studies^5, 12‐23^ that reported on COVID‐19 incubation periods.

**4. DISCUSSION**

The first estimate of the COVID‐19 incubation period was reported by Li et al^12^ as a mean of 5.2 days (95% CI, 4.1‐7.0) and a 95th percentile of 12.5 days (95% CI, 9.2‐18), based on their study on the exposure information of 10 confirmed COVID‐19 patients in Wuhan, China (Table 2). This is slightly longer than the incubation period estimated for SARS, with a median of 4.0 days (95% CI, 3.6‐4.4) and a 95th percentile of 10.6 days (95% CI, 8.9‐12.2).^24^ Subsequent studies^5,12‐23^ on COVID‐19 incubation period (Table 2) reported means varying from 1.8 days to 8.68 days, medians of 4‐8.06 days, and 95th percentiles of 3.2‐16.32 days, which may be due to differences in methodologies and patient samples.

This study of 136 COVID‐19 patients revealed a longer incubation period, with a mean of 8.5 days, an estimated median of 8.3 days and interquartile range 5.3‐11.3 days. Interestingly, two studies^22,23^ also reported longer COVID‐19 incubation periods with mean/median of 8.29/7.76 days and 8.68/8.06 respectively (Table 2). The longer incubation period reported by Qin et al,^22^ who studied a large sample size of 1084 individuals who had been asymptomatic at their time of departure from Wuhan, may be related to the forward follow‐up methodology of a sufficiently long duration of 25 days until symptoms had developed so that those with longer incubation periods were not missed from sample collection. Tindale et al^23^ attributed their finding of longer incubation period to missed intermediate exposure events due to pre‐symptomatic transmission and misperceived exposure times.

Although previous studies on COVID‐19 incubation period did not look specifically at the effect of older age on incubation period, I reviewed these studies for any such clues. Of the twelve studies^5,12‐20,22‐23^ on COVID‐19 incubation period (Table 2) that included adults, nine had information on mean or median age. Except for the first study by Li et al,^12^ which included much older subjects in the parent population but without age information for the 10 patients studied in the incubation period subset, the remaining eight adult studies^5,13,14,16,18‐20,22^ had median age of 40‐52 years with around 15% aged over 65, not too dissimilar to this study population (median age 50.5 years; 16.2% aged over 65 years). However, there was wide variation in the incubation period values reported from these studies (Figure 4), which may be due to differences in methodologies and patient samples. These factors, together with a lack of detailed information on the age structure and the small number of older adults recruited, made direct comparison with this study difficult. Nevertheless, when the clinical and epidemiological study of COVID‐19 by Tian et al^20^in Beijing was reviewed (Figure 4), it was noted that the incubation period for the severe group (whose subjects were older with median age of 61.4 years and 43.5% aged over 65 years) had a longer median incubation period of 7.5 ± 7.2 days when compared with 6.5 ± 4.6 days for the milder group (whose subjects were younger with median age of 44.5 years and 13% aged over 65 years). The study by Qin et al^22^reports on a long COVID‐19 incubation period (mean 8.29 days; median 7.76 days) that is close to the present figures, but their sample was younger (median age 40 years, 13.2% aged over 60 years) and a different methodology was used as discussed above.

**Figure 4:**
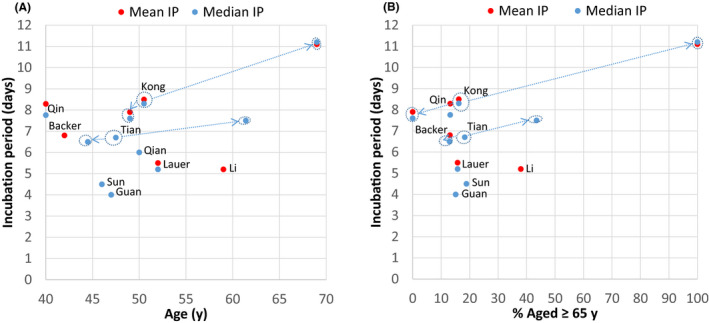
COVID‐19 incubation period (IP) according to age in previous and present studies: (A) mean or median age, (B) percentage aged ≥ 65 years. Substudies are indicated by arrows from main study. “Age” refers to age of study sample of incubation period when available, or to age of parent sample when age is not available for study sample.

**References**

22. Qin J, You C, Lin Q, Hu T, Yu S, Zhou X‐H. Estimation of incubation period distribution of COVID‐19 using disease onset forward time: a novel cross‐sectional and forward follow‐up study. *Sci Adv*. 2020;33:eabc1202. https://advances.sciencemag.org/content/early/2020/08/07/sciadv.abc1202

23. Tindale LC, Stockdale JE, Coombe M, et al. Evidence for transmission of COVID‐19 prior to symptom onset. *eLife*. 2020;9:e57149. https://doi.org/10.7554/eLife.57149

**Table 2:**
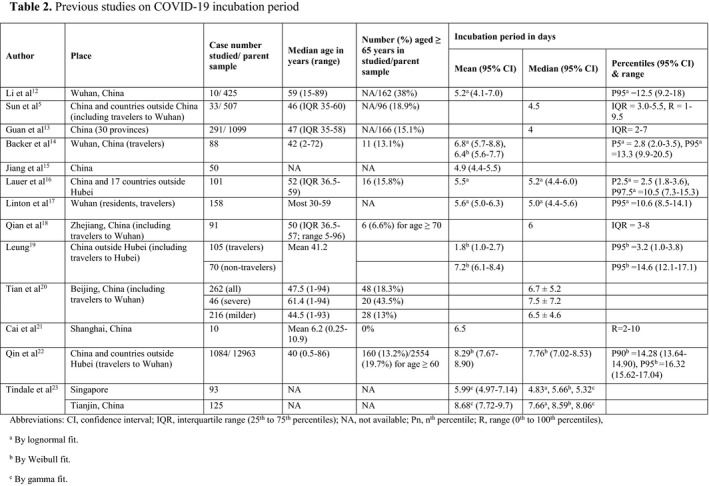
Previous studies on COVID‐19 incubation period
